# Caesarean Scar Pregnancy: A Case Report and a Literature Review

**DOI:** 10.3390/medicina58060740

**Published:** 2022-05-30

**Authors:** George Valasoulis, Ioulia Magaliou, Dimitrios Koufidis, Antonios Garas, Alexandros Daponte

**Affiliations:** 1 Department of Obstetrics & Gynaecology, University Hospital of Larisa, Mezourlo, 41334 Larisa, Greece; gvalasoulis@gmail.com (G.V.); iouliamagaliou@gmail.com (I.M.); dimkoufidis.md@gmail.com (D.K.); garasant@med.uth.gr (A.G.); 2 Hellenic National Public Health Organization-ECDC, Marousi, 15123 Athens, Greece

**Keywords:** caesarean scar pregnancy, caesarean section, scar pregnancy, caesarean scar, caesarean scar pregnancy review

## Abstract

*Background and Objectives*: Caesarean scar pregnancy (CSP) refers to placental implantation on or in the scar of a previous caesarean section and represents a potentially life-threatening condition. CSP is considered a diagnostic challenge in obstetrics, with the diagnosis relying mainly on transvaginal ultrasound (TVS) and the management depending upon case presentation and available healthcare infrastructures. *Case Presentation*: We present a case of 34-year-old G3P2 with a history of two-previous caesarean sections referred to the outpatient gynaecology clinic of our Department at the 7th week (7/40) of gestation with abnormal early pregnancy TVS findings, illustrating the gestational sac attached to the caesarean scar and a foetal pole with evidence of foetal cardiac activity. We discuss the outcome of an alternative combined medical and surgical approach we followed as well as an updated review of the current literature. *Conclusions*: The ideal management of CSP requires tertiary centers, equipment availability and experienced healthcare professionals capable of dealing with any possible complication, as well as individualized treatment based on each case presentation.

## 1. Introduction

Caesarean Scar Pregnancy (CSP) represents one of the rarest forms of endometrial pregnancy [1,2]. Cesarean scar pregnancy is a complication in which implantation situates in the scar from a prior cesarean delivery. Two distinct entities are identified: Type 1 CSP represents implantation on the well-healed scar of the previous caesarean delivery (CD), while in Type 2 the implantation of the placenta takes place within the defect or “niche” of an incompletely healed scar of the previous CD [3]. There is variability in clinical presentation; however, many women present without symptoms. Since diagnosis usually has difficulties and should be done in a timely fashion, fetomaternal subspecialists involvement is necessary for the definition of the final diagnosis and subsequent management of these cases. Transvaginal ultrasonography remains the primary modality for CSP diagnosis, and pregnancy termination is recommended after CSP diagnosis since expectant management was found to be associated with several life-threatening complications that might arise late in the first or in the second trimester, as well as severe maternal morbidity [4]. In our case study presentation, we report a presentation of a patient managed in a tertiary hospital of central Greece and we present a review of the current evidence in the literature.

## 2. Case Report

**Figure 1:**
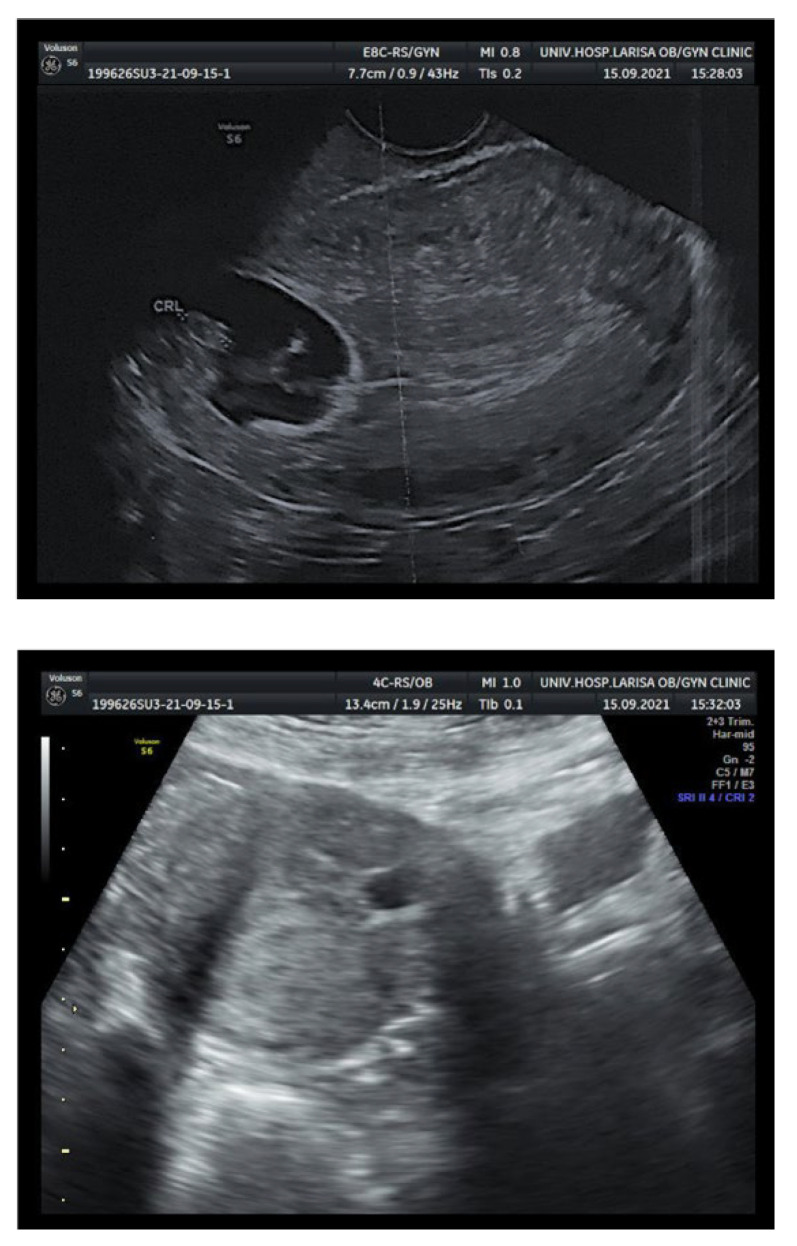
Transvaginal ultrasound showing the gestational sac attached to the caesarean scar.

A 34-year-old gravida 3 para 2 (G3P2) woman with a history of two previous caesarean sections was referred by a physician to the early pregnancy outpatient clinic of the University Hospital of Larisa during the 7th week (7/40) of gestation due to abnormal transvaginal ultrasound (TVS) findings, illustrating the gestational sac attached to the caesarean scar and a fetal pole with evidence of fetal cardiac activity (Figure 1). She had a so far free medical history with no previous surgical operations. The serum levels of beta Human Chorionic Gonadotropin (β-hCG) were elevated (46,407 mIU/mL) at the time of evaluation, and continued to rise sequentially; this particular parameter was considered as a negative prognostic factor [5]. She was asymptomatic with no reported abdominal tenderness, cramping or vaginal bleeding, ruling out a threatened or inevitable miscarriage. On physical examination, she had normal vital signs and cardiovascular and respiratory examination. Her abdomen was soft, palpable and not in tender. The patient was counselled about the potential severity of the condition and all possible life-threatening complications that might arise by an experienced consultant. All possible medical as well as surgical management alternative approaches were discussed in detail and the patient was subsequently admitted.

**Figure 2:**
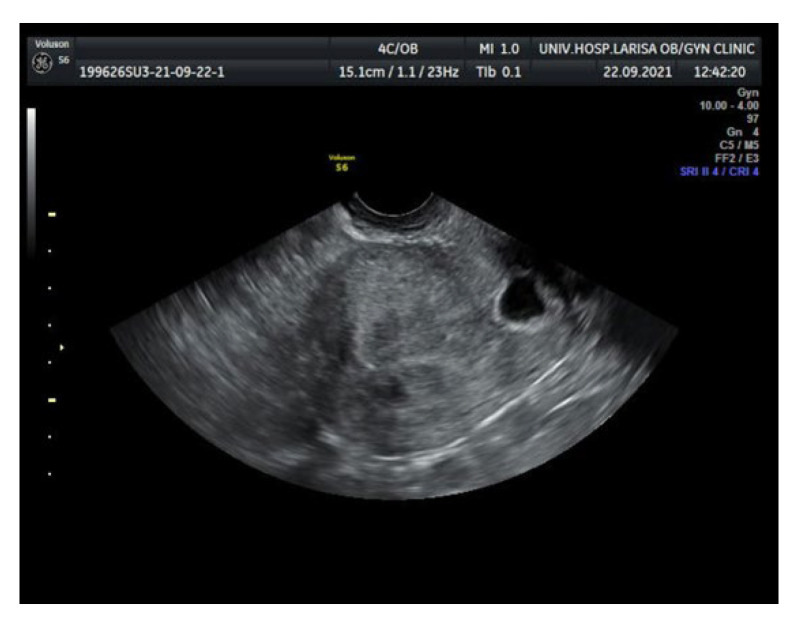
Transvaginal ultrasound 7 days after methotrexate administration.

Initially, after informing the patient accordingly and obtaining written consent, an unsuccessful attempt for pregnancy termination by injecting potassium chloride (KCl) in the gestational sac by ultrasonographic guidance was performed [6,7,8,9]. One day later, despite the elevated β-HCG levels, a decision for medical management with systemic methotrexate (MTX) therapy CSP was made and informed consent was obtained. Based on the protocols, intramuscular administration of a single 8.5 MTX dose (calculated by the equation 50 mg/m^2^) was given [10,11]. Sequential β-hCG levels and TVS were planned [12]. The initial serum levels of β-hCG were 46,407 mIU/mL. Four days after systemic methotrexate administration, β-hCG serum levels continued to rise (52,257 mIU/mL) while a new transvaginal scan revealed a smaller gestational sac with normal shape. On the 7th day after initial MTX administration β-hCG levels reached 52,839 mIU/mL, the patient continued to be asymptomatic, with no vaginal bleeding nor abdominal pain or tenderness and TVS findings remained unchanged (Figure 2).

At that time the medical team considered the possibility of a second dose of methotrexate. However, surgical intervention was favored, and one day later (8th day) decision for a diagnostic hysteroscopy and suction of the scar pregnancy was taken. In particular, suction curettage and foley bulb induction approach was chosen and the patient received counselling and consented accordingly prior to the procedure [13]. The laboratory preoperative bloods and biochemical exams were tested normal, as were renal and liver function assays as well as urinalysis.

### Surgical Procedure

Approximately, two hours prior to the diagnostic hysteroscopy the patient received 800 μg of misoprostol sublingually. Under general anesthesia and ultrasonographic guidance, dilatation of the cervix with Hegars’ was performed and a Karman’s cannula number 4 was inserted to access the sac level via the cervix, followed by suction. Subsequently, a 16 French foley catheter was placed on the sac’s level and the balloon was inflated by saline until the bleeding was stopped, while simultaneously, 10 IU of oxytocin were administered intravenously. The patient recovered with hemodynamical stability and no evidence of intraoperative complications. Twenty-four hours postoperatively the β-hCG levels reached 39,531 mIU/mL. Thereafter, the foley catheter was removed.

**Figure 3:**
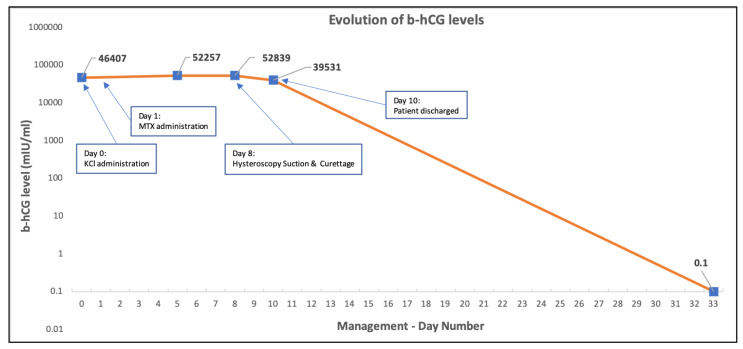
Evolution of b-hCG serum levels since patient’s admission and therapeutic approach.

The patient was discharged from the hospital at postoperative day 2 and one week later histopathological assessment was completed, reporting on products of conception that correspond to a 1st trimester pregnancy and no presence of trophoblastic disease. The β-hCG serum levels were undetectable on the day 24 after surgical intervention (Figure 3).

## 3. Discussion

The first case of a CSP was reported in medical bibliography in 1978 in a G2P1 23 years old South African Zulu woman [14]. As result of the increasing rates of caesarean sections of recent decades, there has been a substantial increase in this gestational pathology incidence resulting in enhanced physician familiarity [1]. The literature reports a varying CSP incidence between 1:1800 to 1:2216 pregnancies with a rate of 0.15% in women with previous caesarean sections, while the incidence is rising in parallel with the number of repeat caesarean sections [13].

The actual mechanism generating this condition remains uncertain. A variety of theories have been proposed so far including: (a) the endogenous migration of the gestational sac through either a wedge defect in the lower uterine segment or a microscopic fistula within the scar [14,15]; (b) invasion of placental villi into the uterine wall at a point of scar dehiscence [16,17,18], and (c) low oxygen tension of scar tissue attracting implantation of the fertilized oocyte [19]. Summarizing the etiology of the particular pathology, a plausible explanations is that CSP could be attributed to defects in the previous formed scar tissue, in terms of microtubular tract development due to poor healing of the trauma caused by procedures such as caesarean section, dilatation and curettage and uterine suction, hysterotomy, myomectomy, abnormal placentation and/or manual removal of placenta as well as in vitro fertilization [20,21,22].

It has to be underlined that scar pregnancy represents a different pathology compared to that of an intrauterine pregnancy with placenta accreta. In cases with placenta accreta formation, the products of conception are primarily settled in the uterine cavity and the leading cause of fluctuating degrees of invasion of the myometrium by trophoblastic tissues is the absence of decidua basalis [21]. In scar pregnancy cases, the gestational sac is completely surrounded by myometrium, and fibrotic tissues of the scar and is separated from the endometrial cavity [21,23]. It is believed that the causing factor prevails the weak vascular support in the uterine front wall in some patients who have undergone caesarean section, where blastocyst implants to the fibrous scar tissue generated by the previous caesarean section and to the myometrium prior to the formation of decidua basalis [24].

Two different types of scar pregnancies have been identified. Type I is believed to be caused by implantation in the scar tissue of the previous caesarean section with expansion towards the cervico-isthmic space or the uterine cavity [22,23]. In this type, a deep implantation in a caesarean scar tissue defect towards the bladder and the abdominal cavity is associated with a higher risk for adverse pregnancy outcomes such as uterine rupture, uncontrollable bleeding, emergency laparotomy and hysterectomy, and maternal morbidity. The second type (Type II) of scar pregnancies refers to implantations growing inside the uterine cavity [21]. Type II scar pregnancies are believed to be caused by deep implantation into scar defect tissues with infiltrating growth into the uterine myometrium, as well as uterine serosal surface, which may result into uterine rupture and massive haemorrhage in the first trimester of pregnancy, with a potential for loss of fertility, when massive haemorrhage necessitates emergency laparotomy and hysterectomy [23]. Symptoms include pelvic pain and first trimester vaginal bleeding; however, many women are asymptomatic at diagnosis [3].

The primary and optimal CSP’s diagnostic modality remains transvaginal ultrasonography in the 1st or early 2nd trimester, which provides high resolution; however, color Doppler in combination to grayscale evaluation is also recommended, allowing detailed visualization of the placental site implantation as well as definition of fetal and extraembryonic structures [5]. The Type I “on-the-scar” or endogenic form, mostly appears to have a considerable ultrasonographic clear layer of myometrium between the anterior uterine wall and the formed placenta. The ultrasonographic features of Type II “in-the-niche” or exogenic form, include a thin myometrial interface below the placenta.

The ultrasonographic diagnostic findings suggestive of CSP may also include: (1) an empty endometrial and endocervical cavity; (2), a nested gestational sac and placenta, on/in the scar; (3) a triangular (≤8/40 weeks), rounded or oval shaped gestational sac (≥8/40 weeks) filling the scar “niche” (the shallow are representing a healed hysterotomy site); (4) a thin (1–3 mm) or absent layer of myometrium between the urinary bladder and the gestational sac; (5) a distinct or rich vascular pattern around the area of the scar, and (6) an embryonic or fetal pole, yolk sac, or both with presence or absence of fetal cardiac activity. Bulging or ballooning of the lower uterine segment in the midline sagittal transabdominal view has also been considered to be supportive of CSP diagnosis [4,5,25] (see Table 1).

In order to assure the maximal benefit from primary diagnosis and treatment, all gestating individuals with a history of previous caesarean sections are advised to be appointed for a first trimester scan at Early Pregnancy Assessment Clinic (EPAC) after a positive pregnancy test. The examination of choice remains transvaginal ultrasonography (TVS), which might be combined with a transabdominal scan in cases where a panoramic view is required, and additional three-dimensional Power Doppler can confirm ultrasonographic impression. In equivocal cases, magnetic resonance imaging (MRI) may corroborate the initial ultrasonographic diagnosis [26,27].

The available treatment modalities comprise of expectant management (an option which has been recently partially opted out by the recommendations of the Society for Maternal-Fetal Medicine—SMFM), medical management with methotrexate administration and surgical intervention; the choice of treatment method is mainly dependent on the case presentation and the clinical symptoms [4]. The available evidence in the literature favors an interventional rather than medical approach based on the success rates, although data are primarily based on case series, as summarized in the recent recommendations by the SMFM [4,28]. In pragmatic terms, clinical manifestations and potential complications are expected and might be expressed more seriously for scar pregnancies. Therefore, surgical intervention combined with any other available approach, ideally individualized, remains the gold standard therapeutic procedure.

Summarizing operative treatment approaches, besides the hysteroscopic, laparoscopic or laparotomic surgical excision, vacuum aspiration and suction, as in our case, can also be used to remove the scar [4,29]. The present CSP treatment modalities include medical management; medical management followed by uterine surgical treatment (usually minimally invasive approach), total laparoscopic hysterectomy (TLH), laparoscopic incision of the uterus and removal of the scar pregnancy foci, vaginal incision of the uterus and removal of the scar pregnancy elements with muscle wall repair of the uterus, as well as selective uterine artery embolization (UAE) [30].

Management of CSP in the first and early second trimesters should preferably be undertaken in a center of excellence where a variety of treatment options and blood bank services are available. All hemodynamically unstable patients should undergo immediate surgical intervention, principally with a minimally invasive approach. For patients with hemodynamical stability, management options include either medical or surgical termination of pregnancy or even continuation of the pregnancy in special circumstances. In CSPs individuals with a fetal demise, expectant management could be offered in combination with medical or surgical treatment [25,31]. Expectant management outcomes appear to be more favorable in patients with Type I (“on-the-scar”) rather than Type II (“in-the-niche”) CSPs, especially in those where thickness of the myometrium is above ≥3 mm [32]. Operative resection of the CSP can be performed laparoscopically, hysteroscopically or with laparotomy [33]. Suction aspiration with ultrasonographic guidance is an alternative CSP approach in the early first trimester (5 to 7 weeks), with additional use of a transcervical balloon catheter in cases where heavy bleeding takes place, as in our case [34]. Transabdominal or transvaginal intragestational injection of MTX under ultrasonographic guidance appears to be an effective treatment option for CSP with success rates as high as 85% in the early first trimester (6 to 8 weeks), however, in more advanced gestations it is difficult to predict the total effectiveness [35]. Transabdominal or transvaginal under ultrasonographic guidance KCl injection (5 mEq into the gestational sac) for a CSP with fetal heart activity has also been described [8]. Medical treatment by systemic MTX use can be administered as an adjunct to all of the above therapies; however, there is not robust evidence supporting this practice [36].

CSP should be treated without delay following diagnosis, and a swift decision for termination should be made because of the increased risk for bleeding in case the pregnancy continues to grow [13]. The principle of the treatment remains to terminate the pregnancy, subtracting the gestational sac, aiming to preserve the patient’s fertility. Currently, most studies indicate that CSP patients with severe type I or type II CSP should receive UAE treatment, which is associated with significant decrease of the risk for developing intraoperative haemorrhage [30].

Only a few cases of laparoscopic treatment of CSP have been reported. Recently, Kathopoulis et al. shared their experience with laparoscopic management of two cases utilizing different operative techniques. While the authors conclude that the laparoscopic approach appears to be a safe and effective technique for the management of CSP, applied either as a primary intervention or after failure of medical management, they consider laparoscopic removal of CSP as mandatory when the scar gestation is growing towards the bladder and abdominal cavity (type II CSP) [37]. Laparoscopic excision of CSP up to 11 weeks of gestation has also been reported [38,39]. The main advantage of the laparoscopic approach is the complete removal of the retained products of conception at the time of the surgery leading to a less prolonged follow-up [13]. Moreover, restored uterine anatomy of the lower segment augments favorable future fertility outcomes [40]. Although representing a reliable treatment approach, it should be performed by skilled laparoscopic surgeons.

A recent meta-analysis by Wu et al. of 32 articles, which were reviewed systematically, including a population of 3380 individuals having CSP history, investigated the reproductive outcomes of women as well as how treatments approaches used might affect subsequent pregnancy outcomes. The authors concluded that these individuals are at higher risk for ectopic pregnancy (16.6%), with recurrent CSP rates and risk for spontaneous miscarriages in cured CSP cases being significantly increased in subsequent pregnancies; therefore, contraception should be recommended in those patients who do not wishing future fertility [4,40]. Based on the available evidence, the authors conclude that any plausible elucidation of how treatment approaches and modalities affect subsequent pregnancies in previous CSP cases seems unfeasible until larger prospective studies are undertaken [40].

## 4. Conclusions

Caesarean scar pregnancy is a rare obstetrical condition, which may result in the woman being in a life-threatening situation such as uterine rupture and massive haemorrhage, possibly leading to maternal death. This situation represents a diagnostic challenge in obstetrics and gynaecology clinical practice, and management should be timely while careful decisions made as soon as possible. Clinicians should rely on transvaginal ultrasonography as the primary diagnostic modality. Women should have access to all appropriate management options for CSP. If local facilities do not provide all options in non-life-threatening situations, then clear referral pathways should exist to allow them to access appropriate care. Ideal CSP management requires specialized centers and blood bank services, appropriate equipment and experienced healthcare professionals who can deal with possible complications. Frequently, management needs to be individualized, as in our case, where decision for combined treatment approaches was made in the concept of personalized medicine and the ideal management of such a life-threatening condition.

## Figures and Tables

**Table 1:** Diagnostic ultrasonographic findings of CSPs.

Empty endometrial and endocervical cavity
Nested gestational sac and placenta on/in the scar
Triangular (≤8/40 weeks) rounded or oval shaped gestational sac (≥8/40 weeks) filling the scar “niche”
Thin (1–3 mm) or absent layer of myometrium between the urinary bladder and the gestational sac
Distinct or rich vascular pattern around the area of the scar
Embryonic or fetal pole, yolk sac, or both with presence or absence of fetal cardiac activity

## Data Availability

Data are available from the corresponding author upon a reasonable request.
